# Factors influencing sanitation and hygiene practices among students in a public university in Bangladesh

**DOI:** 10.1371/journal.pone.0257663

**Published:** 2021-09-22

**Authors:** Ashraful Kabir, Shuvo Roy, Korima Begum, Ariful Haq Kabir, Md Shahgahan Miah

**Affiliations:** 1 Children Without Worms, The Task Force for Global Health, Dhaka, Bangladesh; 2 School of Public Health and Preventive Medicine, Monash University, Melbourne, Australia; 3 Department of Anthropology, Shahjalal University of Science and Technology, Sylhet, Bangladesh; 4 Department of Anthropology, Shahjalal University of Science and Technology, Sylhet, Bangladesh; 5 Institute of Education and Research, Dhaka University, Dhaka, Bangladesh; Helen Keller International, SIERRA LEONE

## Abstract

**Introduction:**

Improved hygiene and sanitation practices in educational settings are effective for the prevention of infections, controlling the transmission of pathogens, and promoting good health. Bangladesh has made remarkable advances in improving higher education in recent decades. Over a hundred universities were established to expand higher education facilities across the country. Hundreds of thousands of graduate students spend time in university settings during their studies. However, little is known about the sanitation and hygiene practice of the university-going population. This study aims to understand and uncover which factors influence students’ sanitation and hygiene behavior in university settings.

**Methods:**

This study was conducted in a public university named Shahjalal University of Science and Technology located in a divisional city of Bangladesh. Based on the Integrated Behavioral Model for Water, Sanitation, and Hygiene (IBM-WASH), we adopted an exploratory qualitative study design. We developed semi-structured interview guides entailing sanitation and hygiene behavior, access, and practice-related questions and tested their efficacy and clarity before use. We conducted seventeen in-depth interviews (IDIs), and four focus group discussions (FGDs, [6–8 participants per FGD]) with students, and seven key informant interviews (KIIs) with university staff. Thematic analysis was used to analyze the data. Triangulation of methods and participants was performed to achieve data validity.

**Results:**

Despite having reasonable awareness and knowledge, the sanitation and hygiene practices of the students were remarkably low. A broad array of interconnected factors influenced sanitation and hygiene behavior, as well as each other. Individual factors (gender, awareness, perception, and sense of health benefits), contextual factors (lack of cleanliness and maintenance, and the supply of sanitary products), socio-behavioural factors (norms, peer influence), and factors related to university infrastructure (shortage of female toilets, lack of monitoring and supervision of cleaning activities) emerged as the underpinning factors that determined the sanitation and hygiene behavior of the university going-population.

**Conclusion:**

The results of this study suggest that despite the rapid expansion of on-campus university education, hygiene practices in public universities are remarkably poor due to a variety of dynamic and interconnected factors situated in different (individual, contextual, socio-phycological) levels. Therefore, multi-level interventions including regular supply of WASH-related materials and agents, promoting low-cost WASH interventions, improving quality cleaning services, close monitoring of cleaning activities, promoting good hygiene behavior at the individual level, and introducing gender-sensitive WASH infrastructure and construction may be beneficial to advance improved sanitation and hygiene practices among university students.

## Introduction

The benefits of improved hygiene and sanitation are well-documented and largely recognized as an effective strategy for the prevention of infection and controlling the transmission of pathogens [1, 2]. The promotion of good hygiene and sanitation practices is also well-recognized as a cost-effective, easy-to-practice, convenient, and useful public health measure to prevent and control the spread of infectious diseases and promote good health [3, 4]. The importance of promoting appropriate sanitation and hygiene practices has been endorsed in many international policy documents and global commitments. The United Nations (UN) emphasized access to improved sanitation and good hygiene practices within the Sustainable Development Goals (SGD target 6), indicating that it is likely to achieve sustainable economic growth and a better future [5, 6].

In recent years, Bangladesh has made overwhelming advances in economic development. The country maintained over six percent of Gross Domestic Product (GDP) over the last two decades and was positioned as the fastest-growing economy in the world [7–9]. Such economic growth enhances the government’s ability to substantially invest in the education sector (i.e., education stipend program, gender parity, geographical coverage), resulting in greater access to school attainment and boosting primary and secondary education [10, 11]. Similar to the primary and secondary education sector, the government also took policy initiatives, such as the promulgation of the first Private University Act in 1992 and a 20-year Strategic Plan for Higher Education 2006–2026. The country has received technical and financial support from the World Bank since the 1990s to expand and meet the demands of higher education in Bangladesh [12]. The Private University Act of 1992 helped to expand higher education in the private sector, whereas the Strategic Plan for Higher Education 2006–2026 focused on the reformation of the entire higher education sector and is largely based on neoliberal policy doctrine [13]. The neoliberal policy shift has emphasized the need to expand technical and market-oriented knowledge [14]. This resulted in the establishment of many science- and technology-oriented new universities in older districts (which have a large population and land territory), headquarters, and townships [13]. According to the most recent statistics of the University Grants Commission (UGC)—the peak body charged with the higher education sector—as of 2020, the country has 148 universities [15]. The UGC’s latest annual report released in 2018 indicated that there are 136 functioning universities (out of 148, the rest are in the process of becoming operational). Of these, 47 are public (state-funded), and 107 are private (non-state-owned) [15]. Over the last decade, students’ enrolment in both public universities and their affiliated colleges and private universities has been increased by ten-fold [15]. Currently, 4,434,451 students are enrolled in a wide array of departments and schools. The share of female students’ enrolment in higher education institutes is around 38 percent [16]. Public universities largely provide on-campus residential facilities in university halls [17]. A high proportion of students stay on campus during their studies, which typically extend over a period of five to seven years. Despite the proven effectiveness of improved sanitation and hygiene practices in educational settings [18], there is evidence that maintaining good hygiene practices in low-income countries has a relatively low implementation rate [19]. Until recently, sanitation and hygiene practice-related studies in educational settings mainly focused on the school level (mostly in primary schools, ages six to eleven) in Bangladesh [20–22]. Few studies have reported the hygiene and sanitation behaviors of university students in the context of Bangladesh. Moreover, most of these studies have used quantitative methodological approach and focused on either the users’ perspective, such as students, or the suppliers’ perspective, such as access to facilities. This is insufficient to completely recognize and explore hygiene and sanitation related behaviour and practices. For example, few cross-sectional studies reported WASH-related descriptive statistics in university students [23–26]. Against this backdrop this paper uses a qualitative methodological approach to investigate hygiene and sanitation related behaviour and practices from a holistic perspective. Adopting a conceptual framework, this study recognizes and explains how and whether sanitation and hygiene practices among university students are influenced. This investigation will provide a total picture of factors associated with WASH-related processes and outcomes in a specific context. This pragmatic study will add information from the perspective of users and suppliers and contribute to the literature. Moreover, the study will inform the Water Sanitation and Hygiene (WASH)-related actions needed in public universities to improve sanitation and hygiene practices among university students.

## Methods

### Study time and setting

This study was conducted at Shahjalal University of Science and Technology (SUST) in Sylhet, a north-eastern city, approximately 240 km from Dhaka, the capital of Bangladesh. SUST is a public university established in 1986 to promote science and technology-oriented education and research. The campus has 320 acres of land [27]. The university supports residential facilities for students in five halls on campus. In addition, the university hires three privately-run halls for its female students outside the campus (in the central city). They are privately managed but aligned with a standard (i.e., WASH facility, security, physical space, etc.) set by the university. Students in these privately-operated halls pay higher rent compared to those who stay in the campus-based halls. In 2020, the university had approximately 11,000 students across seven schools, twenty-seven departments, and two institutes [27]. During their stay on campus, students usually move across different buildings and facilities, including academic buildings, libraries, residential halls, auditoriums, teacher-student center restaurants, cafeterias, and shops. There are several cafeterias, restaurants, and grocery stores that provide essential services for both campus-based students, off-campus students, and visitors. Among these, coffee shops are visited by thousands of students each day for breakfast, lunch, and dinner. There are also many mobile food vendors who prepare and sell various foods across campus [17].

### Theoretical framework

**Fig 1:**
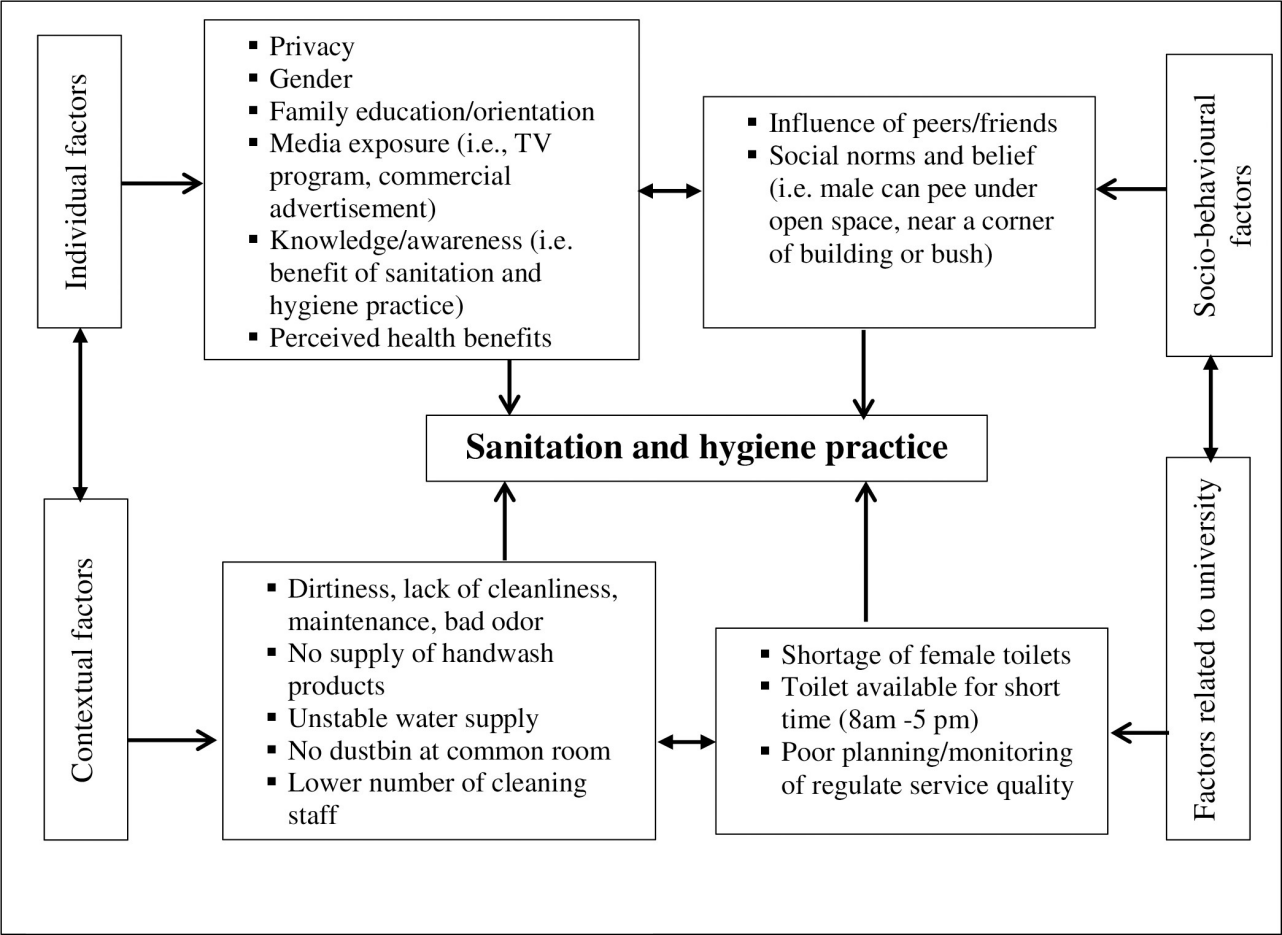
Themes and sub-themes that emerged.

We used the Integrated Behavioral Model for Water, Sanitation, and Hygiene (IBM-WASH) to inform our research [28]. This model offers an analytical and conceptual tool to explore and understand an array of factors that influence the use of water, sanitation, and hygiene dynamically in a resource-limited setting [29]. We, therefore, considered this model the best-suited in the current study setting that is characterized by constraints to the infrastructure required to promote better sanitation and hygiene practices. Based on this model, we developed a conceptual framework to analyze the data (Fig 1). For this model, sanitation and hygiene practices are influenced by a broad range of factors at various levels. The individual-level factors include prior exposure and understanding of the importance of maintaining hygiene. The physical environmental factors refer to the availability of infrastructural facilities or shortages. Socio-behavioural factors are related to the psychological, and social determinants such as norms, beliefs, habits, self-efficacy that influence the adoption of sanitation and hygiene practices. Societal factors related to the institution focus on the polity and/or supply-related issues. The practice of sanitation and hygiene is the combined result of these factors, which are interconnected and influence each other.

### Study population and sampling strategy

We conducted seventeen in-depth interviews (IDIs) and four focus group discussions (FGDs, [6–8 participants per FGD]) with both on-campus (students who stay in the halls) and off-campus students from various departments, and seven key informant interviews (KIIs) with university staff (Table 1). We included undergraduate students based on eligibility criteria such as voluntary participation and completion of at least one year of study. We did not include students who did not have the capacity to provide informed consent or these who were doctoral students or enrolled in short-term courses (evening courses, short professional courses). Considering our study aims, we purposefully recruited the participants, which was used in qualitative studies the context of Bangladesh [30, 31]. First, the interviewers approached potential students and shared the research aims, objectives, and expectations of the study. If the interviewee met all the criteria, they were approached to participate in the interview. During this process, we followed all steps described in the consent form, such as the subject matter of the study, participants’ rights, possible risks and benefits, the liberty of withdrawing from the interview at any point in time, confidentiality, anonymity, and additional information sources. We obtained the written consent of the participants before starting the interview. The interviews were audio-recorded. The number of interviews was determined based on the principle of data saturation; point of time, no new data, or dimension, or theme emerged [32]. Coordinated teamwork throughout had the strength of determining data redundancy, increasing data validity, and rigors. Multiple researchers concurrently conducted and analyzed interviews. After each interview day, the research team completed a debrief sheet to discuss the primary initial/axial codes. After two-thirds of the interviews were completed, the research team checked the independently developed codes and identified that no new information or codes were generated. Following a discussion and having reached a consensus, the research team decided to conduct a few more interviews to reach saturation of directional logic [33]. We stopped interviewing when the data was saturated. However, we considered the three basic principles of selecting a study participant: (i) maximum variation (we included participants from different years or semesters, disciplines, genders, and residences); (ii) iterative process (we re-interviewed two IDI participants and one KII participant to gather relatively accurate and nuanced data by verifying and cross-checking a few pieces of information); and (iii) reflexivity (assessed self-roles).

**Table 1:** Detailed data collection methods and study participants.

Methods	Participant(s)
In-depth Interviews (IDIs) [n = 17, male = 8, female = 9]	IDI1: With on-campus students [stay in the hall/halls] (n = 9); IDI2: With off-campus students [stay in their own house] (n = 8);
Key Informant Interviews (KIIs) [n = 7]	KII1: With cleaners [sweeper] (n = 3); KII2: With hall provost (n = 1); KII3: With house-tutors (n = 3)
Focus Group Discussions [n = 4, each FGD included 6–8 participants (total number of participants; n = 29, male = 15, female = 14)]	FGD1: With on-campus students (n = 2); FGD2: With off-campus students (n = 2)

### Data collection procedure

The data collection team consisted of four members who were graduates in the fields of anthropology and public health. They had extensive expertise in qualitative research methods and the application of different techniques. The second and third authors conducted FGDs, while the first and last authors conducted IDI and KII. The fourth author coordinated the data collection activities with methodological feedback. The interviews were conducted in Bangla, the mother tongue of both the interviewees and interviewers. Semi-structured interview questionnaires and guides were developed and their appropriateness and clarity were tested at another educational institute. These question guides explored several aspects relating to the availability of sanitation and hygiene facilities, facilitators, and/or barriers, habits, infrastructure, supplies, maintenance, and so on. The interviewers first attempted to develop a good rapport with participants by asking about daily activities, preferences, and university-related lifestyles. We subsequently elicited detailed information about sanitation and hygiene-related issues. On average, the IDIs and KIIs took 40–55 minutes and the FGDs 80–100 minutes.

### Data analysis

Adopting this approach, we started to transcribe interviews immediately after completion. After transcribing the data, we translated it into English. We conducted a theme-based analysis following the thematic approach [30, 34]. A stepwise procedure was followed [35]. First, all authors independently and repeatedly read the interviews to familiarize themselves with the content. Second, the primary codes were identified, namely, meaningful statements or information. Third, we searched for some clusters based on similarities in these codes. Finally, we identified four major themes to report the findings (Fig 1). All authors independently prepared some codes and themes to check the similarities and dissimilarities among them. A thematic analysis approach enabled us to develop uncategorized themes, which is useful to explore and capture associated factors that influence sanitation and hygiene practices among students. However, one of the challenges of this approach was organizing and merging the codes/themes that appropriately capture participants’ comments and views. Any disagreements were resolved following a discussion or consensus. No text management software (i.e., Nvivo, Atlas-ti) was used to analyze the data.

### Ethics

This study received ethical approval from the institutional review board of Shahjalal University of Science and Technology. We developed consent forms for participants explaining issues related to confidentiality and anonymity, risk and harm, benefit and loss, additional information sources, and participants’ rights. We maintained all aspects described in the consent form, such as removing any personal identification during the analysis. All data were kept within the research teams. We obtained written consent from the participants.

## Results

The socio-demographic characteristics of the participants are presented in Table 2. The mean age (mean ± standard deviation) of the IDI participants was 20.2 years (20.2±2.4) and 21.6 years for FGD participants (21.6±2.6). The highest number of participants was third-year students in the IDIs (7 out of 17) and FGDs (11 out of 29). The highest number of participants in the IDIs was female (9 out of 17) and from the Faculty of Social Sciences, whereas over half (15 out of 29) were male, and the majority were from the Faculty of Physical Sciences (8 out of 29) for FGD participants. The majority of the participants for both IDIs and FGDs were Muslim and came from rural households.

**Table 2:** Socio-demographic characteristics of the participants (IDI: n = 17, FGD: n = 4).

Characteristics	Methods	
	IDIs (n = 17)	FGDs (n = 4) (each FGD included 6–8 participants [total number of participants; n = 29])
Age in years (mean ±SD)	20.2±2.4	21.6±2.6
**Academic years of study**		
Second years (*n*)	6	10
Third years (*n*)	7	8
Fourth years (*n*)	4	11
**Major of study** (*n*)		
Social sciences (*n*)	5	6
Business studies (*n*)	2	4
Applied sciences & technology (*n*)	4	5
Life sciences (*n*)	2	6
Physical sciences (*n*)	4	8
**Gender** (*n*)		
Male	8	15
Female	9	14
**Family residence background** (*n*)		
Rural area (*n*)	10	19
Urban area (*n*)	7	10
**Religious identities** (*n*)		
Muslim	11	18
Hindu	4	8
Others	2	3

### Thematic analysis

We found that four key factors, namely, individual factors, socio-behavioural factors, contextual factors, and factors related to the university, determine sanitation and hygiene practices among students at the university (Fig 1). Each of these major factors was further divided into sub-factors, as presented below. The data gathered from all sources revealed that students are substantially aware of the need for improved hygiene and sanitation; however, contextual and university-related factors jeopardize their practices.

### Theme 1: Individual and structural factors

Our data revealed that sanitation and hygiene behavior was influenced by a wide array of individual factors, such as level of knowledge and awareness, past habits (developed through family education/orientation, and media exposure), privacy, gender, and the perceived benefits of improving sanitation and hygiene practices. A high proportion of participants were aware of the importance of using sanitary toilets and maintaining personal hygiene. The majority of participants said that they gained a good level of knowledge mostly from their families and schools, such as washing their hands with soap after defecation, and before and after eating, and by coming into contact with dirt throughout their childhoods. One participant stated the following:

"*We normally learned many norms*, *such as ’hand wash behavior’ from our families*. *From childhood naturally*, *mom used to wash my hands after using toilets and obviously before eating food*. *I became used to washing my hands this way*.*" (A male student in an IDI)*

A similar view was shared by another participant:

"*From my childhood*, *my family members taught me how to wash my hands*, *use toilets*, *and maintain good hygiene*. *I think that was the base*. *I am now used to doing so*.*" (A male student in an IDI)*

A parallel view was shared in the focus group discussions:

"*From my childhood*, *my mother trained me how to wash my hands*. *She taught me about healthy sanitation behavior*. *Thus*, *I have adopted a sanitary and hygienic practice*.*" (A female student in an FGD)*

Apart from family, exposure to media (i.e., TV programs, commercial advertisements, and school programs) was reported as a good source of knowledge about sanitation and hygiene habits. One participant explained:

"*I have learned the primary lesson of ’sanitation and hygiene behavior’ from home*. *My mother taught me cleanliness*, *personal hygiene*, *and handwashing from early childhood*. *As I grew up*, *I have learned some additional sanitation stuff (i*.*e*., *liquid soap*, *hand sanitizer*, *sanitary napkins) from my surroundings*, *television programs*, *school wash programs*, *and so on*.*" (A female student in an FGD)*

More than two-thirds of participants said that they felt that using sanitary toilets and maintaining personal hygiene on campus largely depended on how much they allowed them to maintain their privacy. Students viewed toilets and washing facilities in campus buildings including academic buildings, libraries, residential halls, and auditorium buildings as safe places to use. However, female students expressed concern over not having a strict privacy policy. As a result, they emphasized the need to improve privacy issues within campus buildings in toilets and other sanitation facilities. One participant said:

"… *We need privacy*, *and it’s really essential for females*.*" (A female student in an IDI)*

A few students raised concerns about toilets located in places other than university buildings (i.e., located in shopping malls, cafes, restaurants). Toilets in commercial places might not always be safe for women and girls. Thus, the female students felt confident using the toilets and washing facilities in university buildings. Both the male and female participants reported that gender was an issue in toilet use. The participants mentioned that the number of toilets and sanitary facilities is inadequate across university buildings, and that it jeopardized maintaining improved sanitary and hygiene habits, especially for female students. One student reported:

"*There are a lot of problems in campus bathrooms/toilets*. *We have to be in a queue to use the bathroom*, *and it’s quite normal for everyone in the hall*. *To be in a queue might be good in terms of maintaining discipline*. *But suppose*, *if you have an emergency and someone is already in the bathroom*, *then*? *Sometimes things happen like you are in the toilets*, *and you suddenly discover that there is no water*, *then*? *Sometimes you will see there is no soap*, *and toilets are left dirty and uncleaned—these types of things we know happen*, *but still we have to use this kind of dirty and unsafe latrines*.*" (A female student in an IDI)*

Due to the long queues and the unusable conditions of the toilets, female students tended to hold their urine for a long time, which may affect their urinary system. One participant mentioned that holding urine for a long time may weaken the bladder and increase the risk of urinary tract infections:

"*The tendency of holding urine among female students is very high*. *There are many reasons for this*, *including unusable toilet conditions*, *an insufficient number of toilets*, *and unhygienic toilets*. *I guess the incidence of urine infections may be high among female students and this could be linked to holding urine for a long time*.*" (A female student in an FGD)*

Nearly two-third of IDI participants (11 out of 17) shared concerns about the barriers for maintaining menstrual hygiene both in the halls and other buildings. There was no proper arrangement to dispose of menstrual products in the toilets or washrooms. A vast majority of female participants reported that they usually fail to follow hygienic methods for managing menstrual waste. Toilets located in campus buildings were reported as unfriendly to female students as no waste disposal facilities exist except for the presence of a small basket in the toilet area. The following quotes explain the situation:

"*The sanitation system of the campus is not female-friendly*. *Sometimes*, *the supply of water gets stopped for unknown reasons*. *There is no hand wash products or soap in any washroom*. *The only thing present is a small basket and a water pot in some toilets*. *There is no way to flush the toilets after use*. *There should be an emergency pad facility in the female washrooms*, *but unfortunately*, *we cannot see it*.*" (A female student in an FGD)*

"*The scenario of women sanitation facilities on this campus is not good*. *If I have a period of unexpected time*, *I have to go to my room*. *And if I want to change pad*, *there is no dustbin or soap bar or sanitizer*.*" (A female student in an IDI)*

This limitation was acknowledged by the one of the KII participants who said that the gender perspective related to hygiene and sanitation might have been overlooked over the years. The toilets do not seem gender friendly, especially in the academic buildings where no menstrual disposal materials are available. The participant said:

“*The condition is grossly poor*. *But this is worst in the academic buildings which are commonly used*. *Menstrual hygiene management facilities are poor in the academic buildings*.*” (A house-tutor in a KII)*

This view was reflected by another participant:

“*Overall the hygiene and sanitation facilities seem poor for female students*. *However*, *female students may relatively manage this inside the halls*. *But*, *this situation remains poor in academic buildings*.*” (A tutor in a KII)*

The majority of participants reported the perceived benefits of improved sanitation and hygiene practices, which act as a facilitating factor for the adoption of good hygiene habits. However, some female students reported that they carried personal hygiene products (i.e., hand sanitizer, toilet tissue). One participant reported:

"*I carry my own sanitary and hand wash materials*, *so I used that*.*" (A female student in an IDI)*

However, more than two-thirds of the participants reported that a negligible number of students carry personal hygiene materials with them. One participant mentioned:

"*Carrying personal hygiene material has become a good strategy for coping with the situation*, *especially where the resources are limited*. *However*, *this is a recent trend and thus has not been widely practiced*… *it’s quite new in our culture*.*" (A female student in an FGD)*

The majority of male students reported that they do not carry any sanitizer or any other means to maintain hygiene. One participant reported:

*Maybe females and girls are more sensitive and have a good arrangement for carrying any materials. They usually carry a handbag where they easily keep such products. But it is very tough to carry this in the male school bags." (A male student in an FGD)*

### Theme 2: Socio-behavioural factors: Perceptions and beliefs

Mixed views were reported regarding the influence of friends and peers in promoting improved sanitation and hygiene habits. The majority of male participants acknowledged that they were neither motivated nor discouraged by their peers and friends in the adoption of sanitary and hygiene practices. In contrast, female participants said that they sometimes copied or followed what others were practicing. One of the female participants reported:

"*I noticed that some of our friends were carrying personal hygiene products with them*. *I found that it is useful to carry a hand sanitizer or toilet tissue in my bag*… *I can use it if I feel so*.*" (A female student in an FGD)*

Some participants stated that male students were found to urinate in open space mostly at roadsides and in alleyways across campus. In Bangladesh, males often urinate at roadsides or in alleyways; however, it is quite impossible for females to do the same. Unlike female students, male students seemed to have fewer concerns about maintaining hygiene at a personal level. One of the participants stated:

"*I think male students do not think much about sanitation and hygiene issues*. *They get influenced by their surroundings*… *hygiene*, *sanitation*, *may remain concerned*.*" (A male student in an IDI)*

A similar view was shared by a KII informant:

“*In our culture*, *females are more sensitive to maintaining their privacy which may help them adopt positive hygiene and sanitation habits*. *The social and cultural construction support the view that a male person can urinate by a roadside and in alleyways*.*” (A tutor in a KII)*

### Theme 3: Contextual factors

Supply-side factors such as the improper maintenance of toilets, poorly managed services, a lack of cleanliness, unpleasant odors, lack of sanitary products (i.e., soap, hand wash agents, sanitizers, etc.) mostly appeared as constraining factors for the adoption of improved hygiene practice in university settings. The majority of students reported that the cleaning service was insufficient. The university authorities rarely supply soap and/or other sanitary materials. One of the participants stated:

"*I cannot remember any scene that I saw any soap at the toilets*… *the authorities may not be concerned about the importance of providing this product*.*" (A male student in an FGD)*

Additionally, most students reported that their proper hygiene practices are strongly impacted by how well the toilets are maintained and cleaned at the university. Almost all participants said that clogged toilets or dirt were frequently visible in the toilets. Some participants mentioned that the number of toilets was noticeably insufficient, thus creating pressure on the functioning ones. Therefore, regular maintenance of toilets and using sanitary cleaning materials are very critical to maintain cleanliness. However, the reality is, frustratingly, the opposite of this. One participant noted:

"*Toilets are rarely cleaned with proper toilet cleaning products (chemicals)*. *Often*, *they use merely water and/or a small amount of chemical agent which might fail to kill germs*, *remove dirt*, *or clogs*.*" (A female student in an IDI)*

Another student stated:

"*To date*, *inadequate sanitation facilities remain the primary cause of unimproved sanitary and hygiene practices*. *The number of students is huge*, *so the authorities need to provide a good number of hand wash or soaps for the students and take care if they are finished or not*. *The authorities need to appoint more employees to do the cleaning*. *But I hardly noticed that all the mentioned actions were taking place in a timely manner*.*" (A female student in an IDI)*

A similar view was expressed by another participant:

"*The students are aware of the importance of good hygiene habits*. *But often*, *they fail to maintain it mostly because of the supply side*. *You rarely see any handwashing material readily available in the washrooms*. *How can a student maintain good handwashing practices after using the toilet*?*" (A male student in an FGD)*

However, a few KII participants explained why there is such a lack of or shortage in the cleaning workforce and university budget. Nevertheless, they acknowledged that there is a severe shortage of cleaning staff in almost all areas (halls and academic buildings). Thus, there are failures in the routine maintenance of sanitary activities and areas. One participant stated:

"*We don’t have adequate budget support and a workforce to provide sanitary and hygiene support at the optimum level*. *I feel that this causes problems*. *Many of us acknowledge this shortage*, *but it is a very structural problem*.*" (A house tutor in a KII)*

A similar view was shared by a cleaner:

"*The number of cleaners is low*. *It sometimes causes problems in routinely maintaining cleaning activities*. *But I try to do my best*. *The cleaning products are supplied as per rules*.*" (A cleaning staff member in a KII)*

Additionally, some participants reported that unstable water supply triggers the dirtiness or poor maintenance of toilets. On many occasions, the lack of a reliable water supply results in the improper flushing of toilets after use, which deteriorates the toilet hygiene condition. One participant stated:

"*It’s prevalent that one leaves the toilet without proper flushing due to the lack of adequate water*. *This causes the toilet to be unusable for next users*… *thus deteriorating hygiene quality*.*" (A male student in an FGD)*

Another participant noted:

"*Bad odor is very common in the toilets because excreta are not properly flushed out*. *It is created when the toilet holes are not properly flushed out*. *Very often the reason is either a lack of stable water supply or a problem with the flushing system (the flushing system does not work for any reason*.*" (A male student in an IDI)*

### Theme 4: Factors related to the university

Access to toilets and handwashing facilities is restricted even further in the buildings around campus after office hours (8.00 am to 5.00 pm). Regular classes and administrative services are provided during this time; however, students remain on campus for longer periods of time for various reasons, such as library work, group assignments, social gatherings, and so on. During this time, they felt embarrassed as access to the toilets and hand wash facilities remain closed in and around campus. This situation becomes very problematic for female students as they need to go to the nearby halls or restaurants. However, a male student mentioned that it is not unusual for male students to urinate in an open place near a bush or building corner. One participant mentioned:

"*A male student can manage his need by peeing at a roadside*. *But it creates a problem for female students*. *They either hold their needs for a long time or go to the nearby halls*, *which is embarrassing*.*"*

The number of female toilets is noticeably low compared to male toilets, which causes female students to abstain from toilet use. The following participants explained the situation:

"*In our academic building*, *there is only one common room for female students*. *And there are six departments in our academic building*. *So*, *for all the female students*, *there is only one common room*. *In this common room*, *we have only three toilets and one basin*, *which is usable*, *and the other is broken*. *This common room is open till 5 pm*. *But for male students*, *they have two washrooms available in this building*.*" (A female student)*

"*In our academic building*, *there is only one common room for females*, *and it is being rebuilt*. *In the common room*, *there were three washrooms and two basins*, *but one basin was unusable*. *Two new washrooms have been built recently on the ground floor of the building*. *But I am not sure if these new washrooms are for male students*. *There are two washrooms for males and on the opposite side we have two washrooms for females also*.*" (Another female student)*

Some participants mentioned that the lack of appropriate technology and poorly managed construction/repair work made it difficult for students to maintain good sanitary and hygiene practices. A few participants reported that the toilets often do not flush or stop properly, causing the toilet pans to become waterlogged. Some participants mentioned that the authorities (hall administrations, engineering office) do not check the toilets and talk to students about it.

"*I never heard that someone from the authorities talked with students and checked if the cleaning activities are properly maintained*.*" (A male student in an IDI)*

Another participant stated:

"*The toilets are not clean enough to use*. *Though the authorities have employed staff to clean the toilets of the academic buildings and residential halls*, *I don’t think they clean the toilets as required*. *There are bad odors in every toilet*, *and sometimes the toilets seem to be clogged also*. *As I stay in a residential hall*, *I have no other option than using these toilets*. *But I can’t maintain hygiene*, *and I am afraid for my health*. *I think it is about the monitoring and supervision of cleaning work*.*" (A female student in an IDI)*

## Discussion

Using IBM-WASH model, this study aimed to explore and recognize how and which factors influence sanitary and hygiene practices among university students at a public university in Bangladesh. Data from multiple sources and participants identified a broad array of elements situated on different levels of the IBM-WASH model (i.e., individual, socio-behavioural, university, and contextual) that impact sanitation and hygiene practices. These factors are noticeably comparable and interconnected to each other.

In Bangladesh, few studies have examined sanitation and hygiene practices in educational settings. Those that are available tend to focus on primary and secondary schools [36–39]. Beyond the school-based WASH-related studies, few cross-sectional studies have focused on and determined the prevalence of handwashing attitudes, perceptions, and practices in university settings [23, 25]. This shortage of information may limit the scope of comparing and contrasting our results with similar studies. Our findings revealed that university students were aware of and possessed adequate knowledge about sanitation and hygiene-related illnesses and health problems. This contrasts with several international studies in Turkey [40] and elsewhere [41]. The findings of this study revealed that students were aware of the importance of handwashing and possessed positive attitudes towards maintaining good hygiene practices despite structural barriers. These positive attitudes may be linked to WASH-related understanding gained at a pre-university age. Over the past two decades (2000–2020), there has been extensive school-based WASH-related interventions (i.e., hardware facilities such as latrine construction, tube-well installation, supplying handwashing products, cleaning materials, hygiene promotion messaging, IEC materials) in Bangladesh that familiarized and habituated students to adopt good hygiene practices [29, 42–44]. A similar observation was noted in a recent study conducted in Dhaka University, the largest public university in Bangladesh. The study noted that hygiene and sanitation practices differed significantly by gender and socio-economic status; female students and students from nuclear families had better hygiene and sanitation practices compared to male students and students from joint families, where typically three or more generations living together in a single household [25]. Another reason is that university students may come from relatively middle and upper-middle class families with greater access to WASH information (i.e., media exposure) that helps them to adopt acceptable hygiene practices from their families [45, 46]. Another reason may be that the majority of students (mostly born around the beginning of the current millennium) gained contemporary views on lifestyle as informed by (social) media. This is consistent with greater access to telecommunication media associated with economic growth in the last two decades, resulting in improvements in quality of life, particularly to the middle and upper-middle class population. Such views favored the adoption of enhanced sanitary and hygiene practices at personal and family levels. The adoption of enhanced hygiene practices became a symbol of politeness or standard courtesy.

However, our data showed that despite these positive habits related to individual level of WASH behaviors, the lack of contextual and socio-behavioural dimensions of WASH practices at the higher education institutions work as barrier for individuals not to maintain and foster these positive habits at the individual level. The data revealed that students noticeably failed to maintain improved sanitary and hygiene behavior at their university properly. The supply-side response substantially caused this failure, as noted in one study [36]. The university authorities lacked adequate support to monitor and supervise the management of cleaning activities at different levels, which might result in poor outcomes [36]. Similarly, the findings of this study also showed that an inadequate supply of cleaning products (soap, sanitizer, or other means) restricted the timely maintenance quality of sanitation facilities and arrangements across halls and other buildings at the University in Bangladesh. Conversely, the timely and regular supply of sanitary and hygiene material enhanced hygiene practices. A similar observation was noted in systematic reviews and control trials, as well as cross-sectional studies in several international settings [47, 48].

One of the significant findings of this study is that the meaning of sanitation and hygiene was narrowly viewed by participants. Sex and gender sensitivity were largely ignored by the universities in the provision of sanitation-related supplies. The lack of gender sensitivity at the contextual level works as a barrier to improve and foster sanitation and hygienic practices at the individual level. This limitation jeopardized female students adopting improved hygienic behavior in and across campus buildings. Such a lack was noted in Bangladesh and elsewhere in previous studies where girls and women rarely had access to disposing of menstrual products or using cleaning agents [49–52]. Arranging a gender-friendly sanitation system was likely not seriously considered in construction plans. Structural constraints such as reliable water sources, timely managed services, and cleanliness appeared as an underlining barrier for adequate sanitation and hygiene practices. The absence of these factors significantly affected the female students as they are socially and culturally sensitive to using toilets in unfamiliar places (i.e., public toilets). They attempted to take an alternative strategy to cope with such a situation by holding their urine for a long time or tried to avoid drinking water. Due to the study design, our data do not enable us to determine how and whether the female students experienced any health problems caused by this practice. Another study reported that such a strategy negatively impacts girls’ and women’s health outcomes [53–55].

Our findings indicate that institutional-based higher education has expanded rapidly to align with the economic growth of the country in the past two decades. This economic growth might have created increasing demands on hygiene and sanitation facilities. To meet these increasing demands, the Government of Bangladesh announced a national goal known as “Sanitation for All by 2010” as a major policy initiative that facilitated multi-level programmatic supports at household levels [56]. To comply with this national goal, extensive programmatic interventions were implemented at the school level. The need to improve WASH facilities in higher educational institutions was therefore neglected, which resulted in poor facilities, management, and maintenance of WASH facilities. The current study advocates that WASH-related facilities and practices at universities need special focus to promote improved hygiene and sanitation practices in universities.

### Limitation of the study

The findings of this research could not be generalized to those universities that offer fee-earning evening courses. This is because they earn money from professional degrees with high tuition fees and are intended to provide healthy facilities for their professional/executive students (i.e., the Business Faculty or Institute of Education and Research of Dhaka University or private universities). However, the situation of colleges under the National University may be worse than public universities. There are more than 2,200 colleges under the National University offering degrees. Our study is limited as we could not obtain interviews with high-level policy makers/administrators. The high-level policy makers/administrators had engaged with some pre-existing activities during the data collection period that might have focused on sanitation service provision and management in the university. Whilst we included participants from different years, semesters, disciplines, genders, and residences to maximize variation, we recognize that some students, such as those with hearing impairment or other physical/neurological disabilities, were not included in this research, and future research could consider this. However, we are confident that we sampled a diverse range of participants, ensured self-reflexivity, and applied an iterative process during the interviews. Careful consideration of these steps and the standard procedures of qualitative methods enabled us to generate valid evidence that might be generalizable to other public universities.

## Conclusion

This study revealed that sanitation and hygiene practices in public universities are remarkably poor due to supply-side responses. Despite the remarkable increase in the number of universities and resource allocation, the promotion of improved sanitary and hygiene facilities has been overlooked over the years. Therefore, a multi-level promotional intervention focusing on provider responses is needed to advance an enhanced, need-oriented, and effective sanitary and hygiene system that can promote improved hygiene and sanitary practices among university students.

### Suggested recommendations to improve hygiene and sanitation practices

- WASH-related materials and agents (i.e. supplying handwashing products, cleaning materials, washing equipment) should be regularly supplied to ensure quality cleaning services.
- A toilet cleaning checklist may be introduced to ensure quality cleaning services by cleaning professionals.
- WASH-related pictorials and key messages may be developed and displayed to promote good hygiene habits that remind the individual user to maintain good hygiene behavior.
- Regular monitoring and inspection of cleaning professionals and checking the toilet checklist to ensure quality services.
- Promote low-cost solutions such as soapy water or chlorine tablets/a tablet that has been proven effective in resource-limited settings should be introduced to minimize costs.
- Improve provision of toilets for female students. Consider gender perspectives in planning new infrastructure and construction. Low-cost menstrual hygiene-related disposal materials should be placed in existing buildings.

## Supporting information

S1 File Guideline for in-depth interview. (DOCX)

S2 File Guideline for focus group discussion. (DOCX)

S3 File Guideline for key informant interview. (DOCX)
