# Supplementary material for: Factors influencing sanitation and hygiene practices among students in a public university in Bangladesh
Source: PLoS One. 2021 Sep 22;16(9):e0257663. doi: 10.1371/journal.pone.0257663 (PMC8457467; doi:10.1371/journal.pone.0257663)
Supplement: S1 File — (DOCX) [file pone.0257663.s001.docx]

**Guideline for In-depth Interview (IDI): Participants (students across various schools and departments in the university)**

1. Socio-demographic information of the participant (name, sex, age, religion, semester, department, income, residential status of campus, mobile number, email address, etc.)
2. Please tell me briefly about your campus life (i.e., begin with some intro questions like feelings about your university, challenges, and opportunities, etc. faced)
3. Please tell me about your' sanitation and hygiene practice' in the university settings (explore participants' sanitation and hygiene habit/practice at different times, episodes, and locations)?
4. Would you please whether and how you develop your sanitary and hygiene practice? (explore the family background, socio-economical background, exposure to media, etc.) (what, how, why, and why not?)
5. What is the scenario of women's sanitation facilities on this university campus? Does it affect them in maintaining sanitary and hygiene health? (how, and why)?
6. Do you face any challenges/problems/barriers (availabilities of facility, services, knowledge, attitudes, practices, water supply, hand shower/potty/lotta, soap, flies, bad odor, dirty) required for improved sanitary and hygiene practice in the campus? (probe when, how, why, and why not questions).
7. Would you tell us about the good/positive things that motivate you to maintain improved sanitation and hygiene practice? (probe when, how, why, and why not questions).
8. Can you please tell me the overall sanitation system/facilities of this university campus (including academic buildings, libraries, residential halls, etc.)? Does it affect hygiene health) (how, and why)?
9. How and whether sanitation and hygiene practice can be improved in university settings? (what, how, why, and why not?)
