# Supplementary material for: Factors influencing sanitation and hygiene practices among students in a public university in Bangladesh
Source: PLoS One. 2021 Sep 22;16(9):e0257663. doi: 10.1371/journal.pone.0257663 (PMC8457467; doi:10.1371/journal.pone.0257663)
Supplement: S2 File — (DOCX) [file pone.0257663.s002.docx]

**Guideline for Focus Group Discussion (FGD): Participants (students across various schools and departments in the university)**

1. Background characteristics of the participants (name, age, sex, religion, semester, department, income, residential status of campus, mobile number, email address, etc.)
2. Would you please tell us about your experience about sanitation and hygiene practice across the university buildings and settings? (probe when, how, why, and why not questions if relevant).
3. What is the scenario of women's sanitation facilities on this university campus? Does it affect them in maintaining sanitary and hygiene health? (how, why, and why not)?
4. Do students face any challenges/problems/barriers (availabilities of facility, services, knowledge, attitudes, practices, supply sides) in practicing improved sanitary and hygiene habits on campus? (probe when, how, why, and why not questions).
5. Would you tell us about the good/positive things that motivate students to maintain improved sanitation and hygiene habit on campus? (probe when, how, why, and why not questions).
6. What is the overall quality of sanitation and hygiene practices among the students on your campus? (probe when, how, why, and why not questions).
7. Would you please tell me if students received any message/information on sanitation and hygiene behavior on campus? If yes, tell me in detail when, how, who provided?
8. How and whether sanitation and hygiene practices can be improved in university settings? (what, how, why, and why not?)
