# Supplementary material for: Factors influencing sanitation and hygiene practices among students in a public university in Bangladesh
Source: PLoS One. 2021 Sep 22;16(9):e0257663. doi: 10.1371/journal.pone.0257663 (PMC8457467; doi:10.1371/journal.pone.0257663)
Supplement: S3 File — (DOCX) [file pone.0257663.s003.docx]

**Guideline for Key Informant Interview (IDI): Participants (university staff)**

1. Background information of the participants (name, age, position held, workplace, year of services, etc.)
2. Would you please say something about the sanitation and hygiene practice of the students in the university? (What and how, and why do students maintain sanitation behavior? Where and why do students maintain cleanliness? Who and how make sure the healthy sanitation behavior for students? What and how to affect students' sanitation behavior, etc.?)
3. Please say something about your student's toilet use and hand wash. What type of toilet, basin, hand wash materials are availability, shortages, and needs are there? How and when you involved yourself to ensure healthy sanitation behavior? Please discuss elaborately (when, how, why, and why not?)
4. In your opinion, what are the critical elements/aspects that affect students' choices of sanitation behavior in and around your university? (why and why not?)
5. How do these elements/aspects affect student's sanitation behavior and health? (why and why not?)
6. Can you please tell me the overall sanitation system/facilities of this university campus (including academic buildings, libraries, residential halls, etc.)? Does it affect hygiene health) (how, and why)?
7. Would you please share how and whether sanitation and hygiene practices can be improved in the university settings? (what, how, why, and why not?)
